# Can the Honors College’s Top-Notch Training Promote the Deep Learning of Top Innovative Talents?

**DOI:** 10.3390/bs16081352

**Published:** 2026-08-06

**Authors:** Tingzhi Han, Xinyi Fu

**Affiliations:** School of Education, Jiangnan University, Wuxi 214122, China

**Keywords:** Honors College, top innovative talents, deep learning, development

## Abstract

Utilizing comparative analysis and a quasi-experimental design, this study conducted three waves of questionnaire surveys over an 18-month period to longitudinally assess the deep learning levels of students enrolled in a university Honors College, with regular undergraduate students serving as a comparison group. The aim was to examine differences in deep learning development trajectories between the two groups from a dynamic perspective. Results indicated that Honors College students exhibited significantly higher levels of deep learning and greater short-term increases than regular undergraduate students. However, with respect to long-term development, Honors College students did not demonstrate significantly greater overall gains in deep learning, and the observed group differences were not consistently maintained over time. These findings provide insights into patterns of deep learning development among Honors College students and offer references for teaching and management practices in cultivating outstanding innovative talents at Chinese universities.

## 1. Introduction

Since the early 21st century, talent has become a decisive factor in national development and global competition. The Global Talent Competitiveness Index (INSEAD, 2013) further highlights the critical role of skilled and innovative talent in national competitiveness. Against this backdrop, cultivating top-notch innovative talents has become an essential mission for universities, particularly at the undergraduate level. Empirical research has consistently shown that deep learning approaches enable learners to engage in in-depth cognitive processing, stimulate creative thinking, and contribute to the development of innovative capacity (Shaari et al., 2012; Romi et al., 2025; J. L. Zhang, 2020). Therefore, understanding the deep learning trajectories of top innovative talents—reflecting long-term, reflective, and transcendent thinking in a specific professional field—is the starting point of this research.

Since the concept of “top innovative talents” was formally proposed in the 2002 report of the 16th National Congress of the Communist Party of China, the government and Ministry of Education have issued a series of plans. In response to “Qian Xuesen’s Question,” the “Plan for Top-tier Students in Basic Disciplines” (Everest Plan) was launched in 2009 and entered its 2.0 era in 2018. The “Six Excellences and One Top-tier Plan” advanced from 1.0 to 2.0 between 2009 and 2019, and the “Strong Foundation Plan” was issued in 2020 to select students committed to serving major national strategic needs. These plans have contributed to the growing scale of top innovative talent cultivation in Chinese higher education.

As China’s higher education transitions from popularization to universalization, elite education remains indispensable. Trow (1973) noted that mass higher education does not eliminate elite institutions; rather, elite education continues to provide opportunities for highly creative intellectual work. Drawing on Western experiences, many Chinese research universities have established experimental classes, base classes, and Honors Colleges, offering top students high-level education that emphasizes deep learning, intellectual growth, and personality development. Guided by national policies, these institutions aim to prepare students capable of contributing to scientific and technological innovation and social development.

“What is a top innovative talent?” is a key question for universities. The landmark Marland Report (Marland, 1972) defined gifted individuals as those with exceptional performance in intellectual, creative, or academic domains, requiring specialized support to develop their talents. This aligns with China’s concept of “top-notch innovative talents,” who possess outstanding intellectual ability and creative potential. “Innovation” is a key criterion, requiring talents to master traditional knowledge and propose innovative solutions to new challenges. However, identifying innovation potential during university selection (e.g., self-application, written exams, interviews) remains inconsistent and lacks a unified standard.

“How to cultivate top innovative talents?” is another central question. Deep learning—focusing on broader context, internal connections, and knowledge reconstruction—helps learners recall, associate, and reorganize knowledge, facilitating higher-order thinking skills (Hale & Barsalou, 1995). By engaging in deep learning, elite students can explore scientific questions thoroughly, capture essential knowledge logic, and examine problem-solving strategies (Zeng et al., 2025; Egan & Madej, 2009). Monitoring deep learning trajectories has therefore become an important focus for researchers studying talent development.

Global policy studies acknowledge China’s systematic progress in cultivating top-notch talents, including selection, training, resource allocation, and evaluation systems. Supported by tutorial guidance, small-class teaching, and international exchange programs, Chinese universities provide elite students with more advantageous academic environments than conventional undergraduate education. However, comparative studies with the US, UK, and Germany reveal persistent gaps. Relative to mature models, China still faces challenges in institutional maturity, selection procedures, and evaluation mechanisms (W. Zhang & Gao, 2025).

Most existing studies emphasize correlations between investment in educational resources and talent cultivation outcomes, often assuming that high investment is linked to better results. However, student development should be assessed by actual learning outcomes and depth of understanding (Trigwell & Prosser, 1991). Recent research has shifted focus from theoretical discussion and resource allocation to empirical investigation of learning outcomes. Chinese studies have attempted empirical approaches, but the diversity of top-tier cultivation models and overlap with professional training have raised debates about whether observed gains result from student selection or program features.

The deep learning level of top innovative talents is a dynamic process, yet cross-sectional surveys cannot reveal developmental trajectories. Longitudinal tracking provides a way to observe changes in student learning and understanding over time (O. L. Liu, 2011). This study introduces “deep learning” as an observational indicator and applies comparative analysis in a quasi-experimental design, conducting an 18-month, three-round longitudinal survey on Honors College students and ordinary undergraduates. The aim is to examine differences in deep learning development over time, providing insights into student growth patterns and informing teaching and management practices. Given the quasi-experimental design with naturally occurring groups, the findings focus on longitudinal associations and group differences rather than establishing causal effects of Honors College programs.

## 2. Literature Review and Research Hypothesis

### 2.1. The Concept of Deep Learning

Before operationalizing the concept of deep learning, it is necessary to justify why deep learning serves as the focal construct in this investigation, given the availability of alternative theoretical frameworks such as mastery learning, growth mindset, or social contagion in learning communities. Notably, Bartosovic et al. (2023), in designing an introductory physics course, simultaneously incorporated mastery learning, deeper learning, metacognitive awareness, growth mindsets, social contagion, and other pillars as complementary goals (Bartosovic et al., 2023). This integrative approach suggests that these constructs are not mutually exclusive but operate at different levels: mastery learning ensures foundational competency, growth mindset fosters resilience, and social contagion leverages peer effects—yet all ultimately serve to enable deeper learning, which represents the highest-order cognitive outcome. While mastery learning focuses on achieving predetermined standards through repetition and feedback (Bloom, 1968), and growth mindset addresses beliefs about intelligence malleability (Dweck, 2006), neither directly captures the qualitative depth of knowledge integration, critical analysis, and metacognitive reflection that characterize deep learning (Marton & Säljö, 1976). Similarly, social contagion explains emotional and behavioral transmission within peer networks (Bartosovic et al., 2023; Rusticus et al., 2023), but it does not account for how individual learners internally construct meaning from complex content. For top innovative talents in Honors Colleges, whose education aims to cultivate higher-order thinking, creativity, and long-term intellectual autonomy, deep learning provides a more precise and ecologically valid indicator of educational effectiveness. Therefore, selecting deep learning as the core outcome variable allows this study to capture not merely what students know or believe, but how they process, transform, and internalize knowledge—a critical distinction for evaluating elite training programs.

The concept of deep learning was first proposed by Marton and Säljö. As early as the 1970s, Marton and Säljö studied how 30 educational psychology students processed scientific articles and examined their relationship with learning outcomes. From a cognitive psychology perspective, the learning process was categorized into two types: deep processing and surface processing. Surface processing refers to students focusing on memorizing the original content of articles, while deep processing indicates students concentrating on the underlying meaning or core information (Marton & Säljö, 1976). Svensson (1977) identified similar qualitative differences in student learning processes: students adopting holistic approaches primarily focused on authors’ intentions and strived to understand textual meanings, whereas those using atomized methods concentrated on specific details within texts and attempted rote memorization.

Building upon Marton and Säljö’s foundational work, subsequent research has progressively developed systematic theoretical frameworks for deep learning. Entwistle and Ramsden (1983) demonstrated that learners can solve complex problems in unfamiliar contexts by integrating new and existing knowledge, which fully embodies the characteristics of deep learning and profound comprehension capabilities. The academic community continues to explore the mechanisms and influencing factors of deep learning, including learner identity recognition and real-world relevance (Fullan et al., 2017), learning motivation, and teachers’ scaffolding instructional roles (Tochon, 2014). Influenced by the “21st Century Core Competencies” framework, research on outcome-oriented deep learning has gained significant momentum, focusing on cultivating learners’ critical thinking, problem-solving skills, creativity, and communication and collaboration abilities (Asikainen, 2014; Esteban-Guitart & Gee, 2020; Faranda et al., 2021). Ramsden (2003) formally introduced deep learning theory into higher education practice, establishing it as a core objective of quality university teaching and providing crucial guidance for educational applications. From a cognitive science perspective, Bransford et al. (2000) laid the scientific foundation for deep learning, emphasizing that the learning process should activate and reconstruct learners’ existing cognitive structures. Through metacognitive regulation and experiential learning in real-world contexts, knowledge meaning construction is achieved, thereby revealing the essence and operational mechanisms of deep learning. Bloom’s Taxonomy of Educational Objectives, revised by Krathwohl (2002), further elucidates the cognitive hierarchy of deep learning: deep learning corresponds to higher-order cognitive processes such as analysis, evaluation, and creation, while surface learning remains at lower-order cognitive levels like memorization and comprehension. This theoretical framework provides a clear theoretical basis for defining and assessing deep learning objectives.

### 2.2. The Measurement and Assessment of Deep Learning

As a key indicator for measuring student learning quality and innovative talent cultivation, the measurement and evaluation of deep learning have become a central focus in higher education research. Given that deep learning reflects students’ motivation, learning strategies, and persistent characteristics in cognitive processing, self-assessment questionnaires have emerged as the most widely recognized and dominant method for evaluating deep learning—this study focuses on the student population of honor colleges as a representative case study.

Among numerous established and influential assessment tools, Biggs (1987) developed the Study Process Questionnaire (SPQ), which distinguishes three distinct dimensions of the learning process: students’ learning motivation for specific courses and their learning strategies encompassing (1) surface learning, (2) deep learning, and (3) strategy learning. Surface learning refers to meeting basic knowledge acquisition and application requirements through rote memorization; deep learning involves students’ interest in the discipline and their ability to connect new knowledge with existing knowledge; strategy learning encompasses competitive motivation and organized learning approaches. Subsequently, Biggs et al. (2001) optimized this tool by introducing the Revised Study Process Questionnaire (R-SPQ-2F, see details in Appendix A), a two-factor scale with high reliability and validity. This instrument later became the gold standard for measuring deep learning outcomes in higher education, widely applied in international educational contexts such as honors programs and talent development initiatives.

Another prominent approach involves analyzing student assignments (such as papers, projects, or problem-solving tasks) using evaluation criteria specifically designed for deep learning indicators. These criteria typically assess dimensions including critical thinking, knowledge integration skills, metacognitive awareness, and the ability to apply knowledge in new contexts. For instance, Rochford and Borchert (2011) developed structured assessment frameworks through case analysis tasks to evaluate deep learning, focusing on students’ higher-order thinking abilities and problem-solving performance in real-world scenarios. Similarly, in technology-assisted learning environments, Filius et al. (2018) utilized student project submissions and peer feedback materials to measure deep learning engagement in short-term online courses (SPOCs). While this method provides more direct insights into deep learning outcomes, it requires trained evaluators and involves time-consuming implementation processes, particularly evident in large-class teaching settings.

Furthermore, with advancements in measurement methodologies, increasing research efforts have focused on identifying key factors influencing students’ deep learning engagement. These factors encompass individual characteristics (e.g., age, educational background), personal competencies (e.g., learning ability, reflective thinking, communication skills), self-efficacy, teacher autonomy, and emotional support (Groves, 2005; Papinczak et al., 2008; Leung et al., 2012; E. Liu et al., 2022; Zhao & Qin, 2021). Current studies indicate that most assessment tools primarily capture motivational or strategic aspects of deep learning, while few studies comprehensively integrate temporal developmental dimensions. Therefore, longitudinal research remains essential to trace the evolution of deep learning outcomes in specialized educational programs such as Honors Colleges and Elite Talent Development Initiatives.

It should be noted that “deep learning” can be understood at different conceptual levels. In a broad theoretical sense, deep learning is often associated with meaning construction, knowledge integration, critical reflection, higher-order thinking, and creative problem solving. However, the present study does not directly measure all of these broader learning outcomes or innovation-related competencies. Empirically, this study operationalizes deep learning more narrowly as students’ self-reported deep learning approach, measured by the Deep Learning Approach subscale of the R-SPQ-2F. This scale captures students’ tendency to adopt meaning-oriented learning through two dimensions: deep learning motivation and deep learning strategy. Therefore, terms such as creativity, innovation capacity, and higher-order thinking are discussed only as theoretically related outcomes or educational aspirations of honors education, rather than as variables directly measured in this study.

### 2.3. Talent Cultivation and Deep Learning

Honors education plays a pivotal role in higher education talent development. To ensure students not only master disciplinary knowledge and frameworks but also become experts in various fields and lifelong learners, honors programs should guide students toward deep learning methodologies, pursuing meaningful and critical learning approaches rather than mere knowledge repetition (Asikainen, 2014; Biggs, 2003). Honors programs emphasize academic challenges, inquiry-based learning, critical reflection, and integrative thinking—elements that align closely with deep learning principles. Empirical studies demonstrate that participation in Honors College programs significantly enhances students’ deep learning engagement, reflective learning skills, integrative learning proficiency, collaborative learning capabilities, and higher-order thinking abilities (Miller & Dumford, 2018; Seifert et al., 2007). Compared to non-honors students, honors students exhibit more meaning-oriented, exploratory, and deeply processed learning styles, which effectively promote sustained cognitive engagement and knowledge integration (Carnicom & Clump, 2004).

Beyond instructional design, the organizational environment and peer relationship networks within honors education significantly enhance deep learning behaviors. Honors programs foster high-achieving peer communities that provide students with intensive academic interactions, knowledge processing processes, and sustained intellectual engagement (Berger & Milem, 2000). Such learning environments encourage students to transcend surface-level memorization, shifting toward critical thinking, perspective shifts, and meaning construction—as demonstrated by freshmen in honors programs during transformative learning experiences (Knapp et al., 2017). Concurrently, the holistic development model of honors students reveals that academic determination, proactive learning attitudes, and social connectivity work synergistically to effectively strengthen deep learning motivation and promote sustained cognitive engagement (Cuevas et al., 2017). Furthermore, high-impact practices integrated into the honors education system—including undergraduate research projects, global learning experiences, internship opportunities, and practical programs—effectively bridge talent development with deep learning outcomes (Stone, 2025). These structured learning opportunities stimulate proactive exploration, cultivate critical thinking skills, and facilitate knowledge transfer into real-world applications, thereby enhancing deep learning effectiveness. Longitudinal research data further demonstrate that honors-based talent cultivation models consistently improve academic performance and innovation capabilities, with systematic deep learning competencies serving as a key mechanism (Yu et al., 2024). Collectively, these findings conclusively establish honors education as an effective paradigm for achieving deep integration between talent development and deep learning.

Beyond instructional strategies, the cognitive development of top innovative talents can be further understood through neurocognitive models of the mind. Demkanin et al. (2025) applied Tokuhama-Espinosa’s (2019) Theory of the Five Pillars of the Mind—Symbols, Patterns, Order, Categories, and Relationships—to physics education, demonstrating how learners organize knowledge through these domain-specific brain networks. For top innovative talents in Honors Colleges, deep learning is not merely about mastering disciplinary “symbols” or recognizing “patterns,” but about progressing toward higher-order cognitive operations: constructing abstract “categories” and exploring novel “relationships” across disparate knowledge domains. The effectiveness of elite training programs may therefore hinge on whether they deliberately cultivate all five pillars, particularly the transition from lower-order to higher-order pillars, to sustainably foster deeper learning engagement. This theoretical lens offers a promising framework for interpreting the dynamic changes in deep learning observed in the present study.

### 2.4. The Longitudinal Perspective on Deep Learning

Longitudinal studies on learning outcomes emerged as early as the 1970s. For instance, Marton (1975) and Dahlgren (1975) employed pre-post testing designs to examine student learning achievements. Similarly, the groundbreaking research by Marton and Säljö demonstrated longitudinal characteristics. They found that students utilizing deep processing demonstrated enhanced retention of key text information over time (Marton & Säljö, 1976). Conversely, students relying on surface processing could only recall partial textual details in both scenarios, despite receiving relevant cues or correct answers during initial assessments. Although these studies maintained longitudinal frameworks, they solely evaluated learning achievement levels without tracking students’ knowledge processing patterns. Notably, researchers like Svensson (1977) conducted longitudinal studies recording learning data, revealing consistent material processing patterns across time points. However, the proportion of students adopting holistic learning strategies declined, with some transitioning to fragmented learning approaches after the first week.

Following this period, academic interest in research on learners’ cognitive development appeared to stagnate until the mid-1980s, when attention to general learning method development regained momentum. In 1985, Watkins and Hattie pioneered longitudinal measurements of student learning strategies (Watkins & Hattie, 1985). Using the Learning Style Index (ASI), they assessed 540 Australian third-year undergraduates’ learning approaches during their first and third academic years, revealing a declining trend in deep learning methods throughout their studies. However, subsequent research indicated that deep learning strategies did not develop during higher education (Ballantine et al., 2008; Lietz & Matthews, 2010; Rodriguez & Cano, 2007; Zeegers, 2004). Other longitudinal studies also observed a downward trajectory in deep learning approaches (Lietz & Matthews, 2010; Wilding & Andrews, 2006). Additionally, some studies found reduced surface-level learning strategies during higher education (Hall et al., 2004; Gordon & Debus, 2002; Rodriguez & Cano, 2007), while others reported increased surface-level learning patterns (Geitz et al., 2016; Zeegers, 2001). Multiple longitudinal studies have also demonstrated that students’ learning styles remain relatively stable throughout the learning process (e.g., Edmunds & Richardson, 2009; Zeegers, 2001).

Notably, in a 2016 systematic review on Problem-Based Learning (PBL) and deep/superficial learning, Dolmans et al. pointed out that among the 21 empirical studies included, only one employed a rigorous longitudinal tracking design (Dolmans et al., 2016). This study was a five-year longitudinal survey conducted by Reid et al. (2012) targeting medical students, with continuous assessments administered across five undergraduate academic years using the ASSIST scale to measure students’ deep learning, strategic learning, and superficial learning orientations. Results showed that students maintained consistently high overall deep learning levels throughout the five-year program without significant improvement, while superficial learning orientation exhibited only a slight downward trend. The review also included limited quasi-longitudinal or pre-post measurement studies. For instance, Papinczak et al. (2008) found that medical freshmen progressively shifted from deep learning to superficial learning as courses advanced, while Reid et al. (2005)’s multi-round measurements revealed that although deep learning remained at high levels, there was no evident room for growth. These findings demonstrate that deep learning follows non-linear trajectories rather than stable linear progression, with divergent evolutionary patterns across intervention phases. Single cross-sectional measurements cannot fully reveal the true effectiveness of educational models, necessitating long-term tracking studies to capture dynamic changes.

Based on this, the study employs a three-phase longitudinal tracking survey spanning 18 months, which not only captures the dynamic developmental trajectory of deep learning but also systematically evaluates the short-term effects and long-term maintenance outcomes of the Excellence Training Program at the Honors College on deep learning. This approach addresses the limitations of existing research that focuses solely on static characteristics without long-term follow-up.

### 2.5. The Hypothesis of the Study

Building on the dynamic nature of deep learning and integrating the tracking design and comparative approach of this study, we propose the following three theoretical hypotheses:

**Hypothesis** **1.** *Top-notch training programs at the Honors College are associated with significantly greater short-term increases in the deep learning capabilities of top innovative talents within a short period, resulting in substantially higher incremental gains in deep learning compared to regular undergraduates who do not receive elite training.*

**Hypothesis** **2.** *It is hypothesized that after 18 months of continuous top-notch training, the overall developmental increment in deep learning among top innovative talents will be associated with a significantly greater increase compared to that of ordinary undergraduates, suggesting a long-term developmental advantage.*

**Hypothesis** **3.** *It is hypothesized that the top-notch training program at the Honors College will show stable and sustained group differences in deep learning among top innovative talents, maintaining significantly positive differences even during the post-test phase.*

## 3. Methodology

### 3.1. Participants

This study selects top undergraduates from the Honors College of University Jiangnan, a “Double First-Class” university in Jiangsu Province, as the main research objects. To respond to the national call, University Jiangnan established an Honors College in 2009 and launched a “Talent Co-cultivation Model” (quality improvement in the Honors College, professional learning in the professional college). The typical feature of this model is: compared with ordinary undergraduates, these top undergraduates receive professional training from their professional colleges, with the only difference being that they also receive top-notch training from the Honors College. Using ordinary undergraduates as the comparison group, this study examines longitudinal differences in deep learning development associated with Honors College training.

With informed consent, data collection was carried out via online surveys following ethical regulations. The first round was conducted in June 2021, using the self-compiled “Undergraduate Learning Situation Survey” questionnaire for freshmen. Second and third rounds were conducted in March 2022 and December 2022, respectively. Given the attrition of the sample, the effective subjects who participated in all three rounds were 306, 289, and 285. After comparing, the effective sample size for all three rounds was 260, including 135 top undergraduates and 125 ordinary undergraduates. Chi-square and t-tests showed no significant differences in gender ratio, major type, or initial deep learning approach. In summary, the retained sample showed no significant differences in key observed characteristics and was considered suitable for longitudinal analysis.

### 3.2. Research Tools

This study used the self-compiled “Undergraduate Learning Situation Survey” questionnaire to understand the research objects’ personal basic information, college entrance exam information, family background, etc. The Deep Learning Approach subscale of the Revised Two-Factor Study Process Questionnaire (R-SPQ-2F) was used to assess students’ self-reported deep learning approaches. In this study, the measured construct should be understood as students’ tendency to engage in meaning-oriented learning rather than as a direct measure of creativity, innovation capacity, or higher-order thinking performance. The subscale consists of 10 items and includes two dimensions: deep learning motivation and deep learning strategy. The summed score represents the overall level of students’ self-reported deep learning approach. This study conducted three tracking surveys on the same group. After each round of data collection, exploratory factor analysis and reliability tests were conducted. The results showed that the Cronbach’s α coefficients for the motivation dimension were 0.865, 0.898, and 0.941, and for the strategy dimension were 0.817, 0.873, and 0.923. The factor loading coefficients for each item were higher than 0.5, and the Composite Reliability (CR) for each dimension was greater than the acceptable value of 0.7. Thus, the “Deep Learning Approach” scale has high reliability and good structure.

Beyond internal consistency, the validity of the R-SPQ-2F in the Chinese higher education context has been supported by prior psychometric studies (Lu & Li, 2007; Yao et al., 2010; Huang et al., 2013). In particular, Yao et al. (2010) conducted confirmatory factor analysis among 607 Chinese students and confirmed the two-factor structure of the scale with satisfactory model fit, while Huang et al. (2013) reported acceptable reliability and construct validity among Chinese postgraduates. These studies collectively support the use of the R-SPQ-2F in Chinese undergraduate and graduate populations. Accordingly, the results reported in this study refer to changes in students’ deep learning approaches as measured by the R-SPQ-2F.

### 3.3. Analysis Procedures

This study used SPSS 23.0 for data analysis. The specific process is as follows: First, descriptive statistical analysis was performed on the deep learning approach scores and the two sub-dimension scores of top undergraduates in the T1, T2, and T3 stages, and they were classified based on their changes. Subsequently, using ordinary undergraduates as a reference, the Mann–Whitney U test and non-parametric Wilcoxon paired-sample test were used to examine baseline differences, developmental changes, and the stability of deep learning between Honors College students and ordinary undergraduates.

### 3.4. Study Design and Reporting Guidelines

This study employed a quasi-experimental longitudinal design with three rounds of tracking surveys over 18 months. The study adheres to the TREND (Transparent Reporting of Evaluations with Nonrandomized Designs) 22-item checklist for nonrandomized and quasi-experimental studies. The completed TREND checklist has been prepared and will be submitted for editorial and peer review to ensure transparent and standardized reporting.

## 4. Results

### 4.1. Descriptive Changes in Deep Learning Across Three Waves

Table 1 presents the descriptive statistics of the deep learning approach and its two sub-dimensions for Honors College students and ordinary undergraduates across the three survey waves. Overall, both groups showed increases over time, while the Honors College group generally reported higher mean scores than the comparison group.

### 4.2. Primary Analysis: Nonparametric Mann–Whitney U and Wilcoxon Tests

#### 4.2.1. Baseline Comparison at T1

To compare whether there is a difference in the mean value of the total index of deep learning between top undergraduates and ordinary undergraduates in the pre-test stage (T1 stage), this study conducted a Mann–Whitney U test analysis. As shown in Table 2 below, in the pre-test stage, the mean values of the deep learning approach, deep learning motivation, and deep learning strategies of top undergraduates were higher than those of ordinary undergraduates. However, in terms of significant differences in the mean values at the pre-test stage, there were no significant group differences between top undergraduates and ordinary undergraduates in deep learning approach Z = −1.29, *p* = 0.198 > 0.05, deep learning motivation Z = −1.30, *p* = 0.193 > 0.05, or deep learning strategies Z = −0.99, *p* = 0.322 > 0.05.

#### 4.2.2. Difference Test of Stage-Based Increments of Deep Learning from T1 to T2

To test the stage-based difference in deep learning level between the two groups of subjects over 9 months, the deep learning level score in the first post-test (T2) minus the score in the pre-test stage (T1) was used as the stage-based increment index of deep learning level. The Mann–Whitney U test was used to analyze differences in stage-based increments of deep learning between Honors College students and ordinary undergraduates over the 9-month observation period. The research results are shown in Table 3 below. There are significant differences in the stage-based increments of the deep learning approach Z = −2.27, *p* = 0.023 < 0.05, deep learning motivation Z = −2.07, *p* = 0.039 < 0.05, and deep learning strategies Z = −2.21, *p* = 0.027 < 0.05 from T1 to T2 between top undergraduates and ordinary undergraduates. Significant group differences were observed in changes in deep learning from T1 to T2, with Honors College students showing greater increases than ordinary undergraduates.

#### 4.2.3. Difference Test of Overall Increments of Deep Learning from T1 to T3

To test the group differences in the overall increments of deep learning levels of the two groups during the 18 months of receiving elite education, the deep learning level in the second post-test (T3) minus the deep learning level in the pre-test stage (T1) was used as the overall increment. The Mann–Whitney U test was used to compare overall changes in deep learning between Honors College students and ordinary undergraduates over the 18-month observation period. The research results are shown in Table 4 below. The Mann–Whitney U test was used to compare overall changes in deep learning between Honors College students and ordinary undergraduates over the 18-month observation period. No significant group differences were observed in overall increments of deep learning approach (Z = −0.06, *p* = 0.953), deep learning motivation (Z = −0.22, *p* = 0.826), or deep learning strategies (Z = −0.40, *p* = 0.691) from T1 to T3 (see Table 4).

#### 4.2.4. Within-Group Maintenance Test for Honors College Students from T2 to T3

In this study, the first post-test (T2) and the second post-test (T3) were conducted on the deep learning level of top undergraduates at the 9th and 18th months after they received elite training. To test the maintenance effect of top-notch training on the deep learning level of top undergraduates, a non-parametric Wilcoxon paired sample test was conducted on the data of the second post-test and the first post-test. As shown in Table 5 below, the research results show: The deep learning motivation Z=−1.646,p=0.100>0.05 of top undergraduates in the non-parametric Wilcoxon paired sample test between the first post-test and the second post-test was not significant, but their deep learning approach Z=−1.999,p=0.046<0.05 and deep learning strategies Z=−2.097,p=0.036<0.05 differed significantly in the non-parametric Wilcoxon paired sample test between the first post-test and the second post-test. The above research results indicate that top-notch training in the Honors College does not show sustained group differences in the deep learning approach and its deep learning strategy sub-dimension of top undergraduates.

### 4.3. Robustness Analysis: Mixed-Design Repeated Measures ANOVA

To respond to the methodological concern that multiple separate nonparametric tests may not fully model the repeated-measures structure, a mixed-design repeated measures ANOVA was added as a robustness analysis. The model included one within-subject factor, Time (T1, T2, and T3), and one between-subject factor, Group (Honors College students vs. ordinary undergraduates). Deep learning approach was treated as the primary outcome, while deep learning motivation and deep learning strategy were analyzed as secondary outcomes. Mauchly tests showed that the sphericity assumption was not violated for deep learning approach (W = 0.981, *p* = 0.090), deep learning motivation (W = 0.980, *p* = 0.073), or deep learning strategy (W = 0.980, *p* = 0.074); therefore, uncorrected ANOVA results are reported (Table 6).

## 5. Discussion

This study found that the top-notch training program at the Honors College was associated with significantly greater short-term increases in students’ self-reported deep learning approaches over the first nine months, thereby supporting Hypothesis 1. However, over the full 18-month period, the program was not associated with significantly greater overall gains compared with ordinary undergraduates, nor did it show sustained group differences. Thus, Hypotheses 2 and 3 were not supported. These findings suggest that Honors College training may be linked to short-term improvement in deep learning motivation and strategy use, but the present design does not provide sufficient evidence for a sustained long-term advantage. The following explanations should therefore be understood as interpretative reflections rather than direct empirical conclusions, because this study did not directly measure institutional culture, identity formation, peer competition, or students’ subjective experiences of elite education.

First, one possible institutional explanation concerns the tension between the ideals of honors education and its local implementation. Modern university Honors Programs originated in Germany and the United Kingdom and later developed vigorously in the United States (Rinn, 2006). In Western contexts, honors education is often grounded in liberal education and individual autonomy. Liberal education aims to cultivate understanding and critical judgment, whereas over-specialization may reduce individuals to instruments and weaken independent thinking and freedom (Gregory, 2011, pp. 421–423). When an honors education model emphasizing autonomy is introduced into a more administratively structured university setting, its educational ideals may coexist with intensive regulation, assessment pressure, and competitive selection. Based on field observations, fierce peer competition, strict selection and elimination mechanisms, and mandatory extracurricular or practical activities may have contributed to some students’ perceived conflict between autonomous development and mechanical task completion. Such pressures could partly offset the positive learning effects expected from the resource-rich honors environment. The “Talent Co-cultivation Model” adopted by the Honors College in the sample university aims to consolidate students’ disciplinary foundations through professional college education while enhancing their comprehensive qualities through Honors College training. This arrangement may provide students with richer developmental resources. However, the dual academic requirements and assessment standards may also increase students’ workload and reduce the time and energy available for sustained, meaning-oriented learning. This may help explain why the program was associated with short-term gains in self-reported deep learning approaches, but did not produce a clear long-term advantage over the 18-month period.

Second, a possible sociocultural interpretation concerns honor identity. Education not only transmits scientific and cultural knowledge but also shapes students’ understanding of social roles and identities. In Chinese cultural and institutional contexts, educational achievement has long been associated with upward mobility and status recognition. From Bourdieu’s perspective, status distinctions are closely related to unequal access to symbolic and organizational resources, and such distinctions become an important source of symbolic meaning (Bourdieu, 1984). In this sense, the Honors College may provide top students with a distinctive identity symbol, which can strengthen positive self-recognition and a sense of belonging. However, if honor identity is interpreted mainly as status distinction, it may also encourage some students to focus on maintaining elite status rather than engaging in sustained inquiry, reflection, and meaning-oriented learning. This interpretation should be treated cautiously, because the present study did not directly assess students’ identity perceptions, symbolic consumption, or subjective experiences of honors status. Future qualitative or mixed-methods research is needed to examine whether and how honor identity, peer competition, and institutional culture influence students’ motivation, self-regulation, and long-term engagement in deep learning approaches.

## 6. Conclusions and Implications

This study finds that Hypothesis 1 is supported, whereas Hypothesis 2 and Hypothesis 3 are not supported. The Honors College’s top-notch training only shows a significant short-term group difference in deep learning, but has no obvious long-term advantage or sustained group difference. This does not mean that a long-term advantage is unattainable; rather, it suggests that such an advantage may depend on whether Honors Colleges can transform short-term resource and identity advantages into sustained developmental mechanisms. Therefore, the following suggestions are proposed not merely as practical recommendations, but also as hypothetical pathways through which Honors Colleges may convert temporary gains in deep learning into long-term advantages: reducing excessive regulatory pressure, strengthening personalized academic support, reconstructing honor identity as responsibility, and establishing developmental evaluation systems that continuously reinforce intrinsic motivation, metacognitive regulation, and knowledge integration.

First, Honors Colleges should shift from rigid regulation to personalized cultivation. From the perspective of the “Five Pillars of the Mind” theory (Tokuhama-Espinosa, 2019; Demkanin et al., 2025), the shift from “rigid regulation” to “personalized cultivation” requires elite training to move beyond symbol recognition and pattern application toward higher-order capacities such as abstract categorization and cross-disciplinary connection. The short-term gains found in this study may suggest that current honors education enhances basic cognitive skills but provides limited autonomy and scaffolding for sustained deep learning and innovation. Thus, personalized cultivation should systematically support higher-order cognitive development. To support top innovative talents, universities should provide psychological support, emotional care, and individualized guidance to help students cope with academic pressure and peer competition. Professional counseling services, regular mental health education, and structured peer activities can reduce stress, strengthen students’ sense of belonging, and foster a supportive learning environment. Meanwhile, Honors Colleges should improve the tutor system so that mentors can better identify students’ developmental needs, provide academic scaffolding, and offer timely resource support. In addition, a diversified, process-oriented evaluation system should be established to assess students’ development through multiple dimensions, including academic performance, project outcomes, research engagement, and innovation potential, rather than relying solely on examinations and grades. If implemented consistently, these measures may reduce the emotional costs of elite education, sustain students’ deep learning motivation and strategy use, and create conditions under which short-term improvement can gradually develop into a long-term developmental advantage.

Second, Honors Colleges should guide students from the symbolic consumption of honor toward a responsible understanding of honor identity. Peirce’s semiotic theory provides a useful lens for understanding how honor functions as a symbol. According to Peirce (1867–1893/1992), a symbol involves a dynamic relationship among the representamen, object, and interpretant; its meaning is not fixed but is continuously interpreted and reconstructed in specific social contexts. Thus, the meaning of “honor” may vary across students and institutional settings. When honor, titles, or elite status are pursued mainly for symbolic distinction rather than intrinsic educational value, a tendency toward symbolic consumption may emerge (Veblen, 1899). In the context of Honors Colleges, this may lead some students to focus excessively on status recognition while neglecting the academic responsibility and developmental mission embedded in honors education. Therefore, Honors Colleges should help students reinterpret honor identity as a source of academic mission, social responsibility, and commitment to public value. Honors education should encourage talented students to align personal development with national needs, human development, intellectual innovation, and social welfare (VanLaningham et al., 2019). If honor identity is reconstructed in this way, the symbolic advantage of being an Honors College student may be transformed into a more durable sense of responsibility and self-regulated learning. Such a transformation may, in turn, support long-term deep learning by shifting students’ attention from maintaining elite status to sustained inquiry, reflection, and knowledge creation.

Taken together, the proposed reforms are expected to operate through a sequential mechanism: institutional flexibility and emotional support reduce pressure and maintain learning engagement; personalized mentoring and process-based evaluation strengthen metacognitive regulation and knowledge integration; and responsibility-oriented honor education transforms external recognition into intrinsic academic commitment. Although these pathways remain hypothetical and require further empirical testing, they provide a clearer explanation of how implementing the above suggestions could help Honors Colleges move from short-term improvement toward a sustainable long-term advantage in deep learning development.

## 7. Limitations and Future Research

Several limitations should be acknowledged.

First, the R-SPQ-2F measures students’ self-reported deep learning approaches, including motivation and strategy, but does not directly assess creativity, innovation capacity, higher-order thinking performance, or transformative intellectual development. Therefore, the findings should be interpreted as changes in reported deep learning approaches rather than direct evidence of broader innovation-related competencies. Future research should combine self-report scales with performance-based assessments, project evaluation, research outputs, and qualitative evidence.

Second, this study did not conduct formal longitudinal measurement invariance testing. Although the R-SPQ-2F showed acceptable reliability and factor structure across the three waves, future studies should use multi-wave confirmatory factor analysis to test configural, metric, and scalar invariance before making stronger claims about developmental change.

Third, the analysis did not include objective academic indicators, such as GPA, course grades, research outputs, or project performance, nor did it control for potentially relevant background factors, such as prior academic achievement, socioeconomic status, family educational resources, personality traits, or initial learning motivation. Future research should incorporate richer covariates and objective performance indicators to provide a more rigorous assessment of Honors College education.

Finally, the sociocultural explanations concerning symbolic identity, elite education, and Confucian traditions should be understood as interpretative reflections rather than conclusions directly demonstrated by the quantitative data. Future research should use interviews, observations, learning portfolios, or mixed-methods designs to examine these mechanisms more directly.

## Figures and Tables

**Table 1:** Descriptive statistics by group and survey wave.

	Group	*n*	T1 M (SD)	T2 M (SD)	T3 M (SD)
Deep Learning Approach	Honors College	135	33.79 (7.25)	35.67 (8.60)	37.15 (9.00)
	Comparison	125	32.86 (9.36)	32.21 (9.42)	35.95 (10.31)
Deep Learning Motivation	Honors College	135	17.00 (4.11)	17.99 (4.52)	18.68 (4.61)
	Comparison	125	16.41 (4.86)	16.17 (4.80)	18.14 (5.13)
Deep Learning Strategy	Honors College	135	16.79 (3.55)	17.69 (4.30)	18.47 (4.52)
	Comparison	125	16.46 (4.66)	16.04 (4.77)	17.81 (5.28)

Note. Comparison Group = ordinary undergraduates without top-notch training.

**Table 2:** Difference test of deep learning level between two groups of undergraduates at pre-test.

	Group	M (SD)	Mean Rank	U	Z	*p*
Deep Learning Approach	Honors College	33.79 (7.25)	136.27	7659	−1.29	0.198
	Comparison	32.86 (9.36)	124.27			
Deep Learning Motivation	Honors College	17.00 (4.11)	136.32	7651.5	−1.30	0.193
	Comparison	16.41 (4.86)	124.21			
Deep Learning Strategy	Honors College	16.79 (3.55)	134.93	7840	−0.99	0.322
	Comparison	16.46 (4.66)	125.72			

**Table 3:** Difference test of stage-based increments of deep learning level between two groups of undergraduates.

	Group	M_T2−T1_ (SD_T2−T1_)	Mean Rank	U	Z	*p*
Deep Learning Approach	Honors College	1.88 (9.12)	140.67	7064.5	−2.27	0.023
	Comparison	−0.66 (10.41)	119.52			
Deep Learning Motivation	Honors College	0.99 (5.00)	139.76	7188	−2.07	0.039
	Comparison	−0.24 (5.28)	120.50			
Deep Learning Strategy	Honors College	0.90 (4.58)	140.39	7102.5	−2.21	0.027
	Comparison	−0.42 (5.40)	119.82			

**Table 4:** Difference test of overall increments of deep learning level between two groups of undergraduates.

	Group	M_T3−T1_	SD_T3−T1_	Mean Rank	U	Z	*p*
Deep Learning Approach	Honors College	3.36	9.38	130.76	8402	−0.06	0.953
	Comparison	3.09	12.49	130.22			
Deep Learning Motivation	Honors College	1.68	5.09	129.51	8304.5	−0.22	0.826
	Comparison	1.74	6.30	131.56			
Deep Learning Strategy	Honors College	1.67	4.71	132.28	8197	−0.40	0.691
	Comparison	1.35	6.38	128.58			

**Table 5:** Difference test of two post-tests of deep learning for top undergraduates.

	First Round Post-Test		Second Round Post-Test		Z	*p*
	M	SD	M	SD		
Deep Learning Approach	35.67	8.60	37.15	9.00	−2.00	0.046
Deep Learning Motivation	17.99	4.52	18.68	4.61	−1.65	0.100
Deep Learning Strategy	17.69	4.30	18.47	4.52	−2.10	0.036

**Table 6:** Mixed-design repeated measures ANOVA results.

	Effect	df1	df2	F	*p*	Partial Eta Squared
Deep Learning Approach	Group	1	258	4.87	0.028	0.019
	Time	2	516	14.43	<0.001	0.053
	Group × Time	2	516	2.41	0.091	0.009
	Group	1	258	5.06	0.025	0.019
Deep Learning Motivation	Time	2	516	14.61	<0.001	0.054
	Group × Time	2	516	2.39	0.093	0.009
Deep Learning Strategy	Group	1	258	4.37	0.037	0.017
	Time	2	516	12.66	<0.001	0.047
	Group × Time	2	516	2.25	0.107	0.009

## Data Availability

Data are available on request due to privacy and ethical restrictions.

## Appendix A

**R-SPQ-2F:**

1. I find that at times studying gives me a feeling of deep personal satisfaction.
2. I find that I have to do enough work on a topic so that I can form my own conclusions before I am satisfied.
3. I feel that virtually any topic can be highly interesting once I get into it.
4. I find most new topics interesting and often spend extra time trying to obtain more information about them.
5. I find that studying academic topics can at times be as exciting as a good novel or movie.
6. I test myself on important topics until I understand them completely.
7. I work hard at my studies because I find the material interesting.
8. I spend a lot of my free time finding out more about interesting topics which have been discussed in different classes.
9. I come to most classes with questions in mind that I want answering.
10. I make a point of looking at most of the suggested readings that go with the lectures.

**Table A1:** TREND 22—Item Checklist.

No.	TREND Item	Completed (✓)	Description/Answer
1	Title: Identify the study as nonrandomized in the title	✓	Can the Honors College’s Top-notch Training Promote the deep learning of Top Innovative Talents? A Quasi-Experimental Longitudinal Study
2	Abstract: Structured abstract including objectives, design, methods, results, conclusions	✓	Standard structured abstract provided
3	Introduction: Scientific background and rationale for the intervention/evaluation	✓	See Section 1
4	Intervention: Clear definition of the intervention (top-notch training program)	✓	Honors College top-notch training (curriculum, tutorial system, interdisciplinary practice)
5	Study design: Specify nonrandomized design type (quasi-experimental)	✓	Quasi-experimental with three-time longitudinal tracking
6	Participants: Description of study participants (eligibility, setting, location)	✓	Undergraduates from a Double First-Class university in Jiangsu; 135 elite students, 125 ordinary students
7	Recruitment: How participants were recruited and selected	✓	University enrollment and Honors College selection
8	Nonrandomized assignment: Method used to assign groups (non-random)	✓	Natural grouping: Honors College elite students vs. regular undergraduates
9	Baseline: Data on baseline characteristics of groups	✓	See Table 1 (pre-test T1 showed no significant difference)
10	Outcome: Primary and secondary outcome measures clearly defined	✓	Primary: Deep learning approach; Secondary: Deep learning motivation, deep learning strategy
11	Outcome measurement: When and how outcomes were measured	✓	Three waves: June 2021 (T1), March 2022 (T2), December 2022 (T2); R-SPQ-2F scale
12	Sample size: Description of sample size determination	✓	Total effective N = 260; balanced group comparison
13	Statistical methods: Statistical methods used for analysis	✓	Mann–Whitney U test, Wilcoxon signed-rank test, descriptive statistics
14	Participant flow: Flow of participants through the study	✓	Three rounds; 260 completed all waves
15	Recruitment period: Dates of recruitment and data collection	✓	June 2021–December 2022 (18 months)
16	Baseline comparability: Comparison of baseline characteristics	✓	No significant difference at T1 (*p* > 0.05)
17	Results for each group: Outcomes for each group reported	✓	See Section 4.1, Section 4.2 and Section 4.3
18	Effect size: Estimate of effect size with precision (e.g., 95% CI)	✓	Reported Z and *p*-values; mean differences provided
19	Missing data: Handling of missing data and attrition	✓	Only complete cases included; minimal attrition
20	Ancillary analyses: Any subgroup or sensitivity analyses	✓	No additional subgroups
21	Limitations: Study limitations discussed	✓	See Section 5
22	Interpretation: Interpretation of results consistent with findings	✓	See Section 5 and Section 6
